# Fluoride Content, Availability, and Stability in a Propolis-Based Dentifrice

**DOI:** 10.1155/ijod/3414733

**Published:** 2025-04-10

**Authors:** Mara Assef Leitão Lotif, Lidia Audrey Rocha Valadas, Peter Bottenberg, Aldo Squassi, Thereza Cristina Farias Botelho Dantas, Vanara Florêncio Passos, Edilson Martins Rodrigues Neto, Mary Anne Medeiros Bandeira, Marta Maria de França Fonteles

**Affiliations:** ^1^Pharmacy Department, Pharmacy, Dentistry and Nursing College, Federal University of Ceara, 1210 Pastor Samuel Mulunga Street 60430372, Fortaleza, Ceara, Brazil; ^2^Universidad de Buenos Aires, Facultad de Odontología, Cátedra de Odontología Preventiva y Comunitaria, Buenos Aires, Argentina; ^3^Dental School, Free Univeristy of Brussels (ULB), Brussels, Belgium; ^4^College of Dentistry, Paulo Picanço College, Fortaleza, Ceara, Brazil; ^5^College of Dentistry, Christus University Center, Fortaleza, Ceara, Brazil

**Keywords:** dental caries, dentifrices, natural products

## Abstract

**Purpose:** This study aims to verify the stability of different types of fluoride in a dentifrice containing Brazilian red propolis (BRP).

**Methods:** The dentifrice formulation was developed with calcium carbonate (CaCO_3_) and sodium monofluorophosphate (MFP) (patent INPI BR1020170110974). Nominal fluoride content was 1500 μg/g. Batches of five lots were selected and analyzed for 2 consecutive years (fresh, with 12 and 24 months). Dentifrices from each tube were analyzed in duplicate using an ion-specific electrode (ISE). The concentrations of fluoride (total fluoride [TF], total soluble fluoride [TSF], and ionizable fluoride [IF]) were determined, and the results were expressed in ppm F (μgF/g).

**Results:** After 12 months of aging, the samples showed TF concentration ranging from 1198.9 ± 26.9 to 1443.6 ± 33.4 ppm F, TSF ranging from 869.6 ± 105.5 to 965.8 ± 149.8 ppm F, and IF ranging from 223.5 ± 14.8 to 269.7 ± 10.1 ppm F. After 24 months of aging, the samples showed TF concentration ranging from 763.5 ± 73.2 to 1083.1 ± 32.4 ppm F, TSF ranging from 552.3 ± 39.9 to 758.4 ± 141.1 ppm F, and IF ranging from 102.2 ± 4.0 to 174.7 ± 4.9 ppm F.

**Conclusion:** Soluble fluoride remained relatively stable until 12 months of aging; however, at 24 months of aging, the concentration reduced.

## 1. Introduction

The incorporation of natural products in oral care formulations has been an alternative to traditional active ingredients [1, 2]. Resistance to synthetic antimicrobials and the search for substances with biological properties with less adverse effects have increased interest in natural products [3–5]. The high demand and investment in biotechnology of products derived from bees have contributed to launching several products on the market, such as those incorporated with propolis [3, 6].

Propolis is a complex, nontoxic resinous mixture which is collected by bees of the *Apis mellifera* species from different parts of plants that are associated with the salivary secretions and enzymes of these bees, resulting in this material that is rich in biological properties. This complex mixture is used by bees to seal the hive and for its asepsis action through its antimicrobial properties. Propolis has been used for centuries in traditional medicine to treat oral and systemic diseases due to its biological activity and quantities of chemical constituents [7, 8].

There is strong evidence regarding the therapeutic effect of propolis extract on various microorganisms in dental biofilm, in addition to presenting itself as a low toxicity clinical option [9, 10]. The number of patents has been increasing, and this product is used in several formulations such as capsules, extracts, gels, dentifrice, sprays, and mouthwash, among others. Some of the properties of propolis includes, antibacterial, anti-inflammatory, antifungal, antiviral, antioxidant, and antitumor properties [7, 11, 12].

The Brazilian red propolis (BRP) is found in Alagoas in the northeast of Brazil, known for the red coloring coming from the pigments of the local plants, and specifically *Dalbergia ecastaphyllum*, which has its biological origin in this region [8, 13]. This type of propolis is exclusive to this region, having a high concentration of isoflavonoids in its composition, which in turn led to the Brazilian National Institute of Industrial Property (INPI) granting the title of geographical indication for this location, ensuring an international certificate of being the sole producer of this type of propolis in the world with its quality assured throughout the year [7, 9, 10, 14, 15].

Dentifrices with antimicrobial bioactive molecules have been widely studied and commercialized. It is expanding in production and consumption in the international market, in addition to an increasing modernization of derivative products and growing interest in the standardization of products [7, 16, 17]. Among dental products with propolis, patents about dentifrices have stood out in recent years due to the product's antimicrobial and anti-inflammatory action [11].

However, in addition to these properties, the presence of fluoride is essential in order to control and prevent tooth decay, with this element being the most evident in relation to tooth decay. It is believed that fluoridated dentifrices are considered the main reason for the decline in the prevalence of caries in recent decades [18, 19] and are considered the most accessible form of fluoride delivery [20–23].

The BRP dentifrice in the present study has already demonstrated clinical and microbiological efficacy in treating gingivitis in orthodontic patients, reducing the marginal bleeding rate and the colony count of *Streptococcus mutans* and gram-negative bacteria [13]. However, quality control must ensure appropriate fluoride concentrations which maintain the anticaries potential of the formulation. For a dentifrice to be effective in controlling tooth decay, an adequate concentration of fluoride in soluble form must be present in the formulation, such as fluoride ion like monofluorophosphate (MFP). Only fluoride in soluble form can interfere in the dynamic process of caries, reducing demineralization and favoring the remineralization of dental hard tissues [18, 24, 25]. Thus, dentifrices must have a concentration of at least 1000 ppm of soluble fluoride to have the maximum potential to interfere with the caries process [19, 26, 27].

The need to evaluate total soluble fluoride (TSF) is essential because even though the total fluoride (TF) content (MFP) of the dentifrice is high, it is important to note that fractions of fluoride may be insoluble, such as precipitated CaF_2_. Products formulated with MFP and calcium-based abrasives (such as the present formulated dentifrice) tend to precipitate CaF_2_ over time, and this is also influenced by storage conditions, which decreases the amount of the soluble form. Therefore, the TF content must be increased up to 1500 ppm F in these cases [28].

Dentifrices with antimicrobial molecules are important to oral care, but it is important to assure the anticaries activity and the remineralization process. Thus, the objective of the present work was to analyze fluoride concentrations in different forms in an experimental BRP dentifrice (patent number INPI BR1020170110974) in order to verify the availability of active fluoride found in the product in fresh and aged samples.

## 2. Material and Methods

### 2.1. BRP Extract and Dentifrice Preparation

The red propolis extract was collected from the city of Marechal Deodoro (south latitude 9°44.555', west latitude 35°52.080', and altitude of 18.1 m above sea level), a region with a geographical indication granted by the INPI, in the state of Alagoas, Brazil. The use of the extract for this study was authorized by regulatory agencies for the control of Brazilian Genetic Heritage and Biodiversity Conservation under protocol number A854028.

The extraction was carried out by maceration in 80% ethanol to obtain an ethanolic extract of BRP. Liquid–liquid extraction of this crude extract was performed to eliminate grease and waxes. The crude extract (80 g) was solubilized with absolute ethanol (350 mL), and then 150 mL of distilled water was added followed by vigorous agitation for 2 min. That extract was transferred to a separation funnel, and hexane (500 mL) was added to eliminate the grease and wax. The hexane layer was removed with a separation funnel, and then ethyl acetate solvent was added in two liquid–liquid extraction steps to obtain an ethyl acetate extract enriched with flavonoids and isoflavonoids from BRP, free of grease and wax [29].

BRP extract at 1% concentration (previously studied antimicrobial concentration) was incorporated into the fluoridated dentifrice (1500 ppm MFP) in the pharmaceutics laboratory of the pharmacy course of the Federal University of Ceará, Brazil. The identification of the constituents was performed by high-performance liquid chromatography (HPLC) (Shimadzu, Kyoto, Japan) equipment with CBM-20A controller, LC-20AT quaternary pump, diode array detector SPD-M 20A, and Shimadzu LC version 1.21 SP1 software. Markers of BRP were identified with the main constituents of quercetin, vestitol, and neovestitol. Identification was performed by comparing the chromatographic profile of the BRP samples in relation to the standards of the isolated chemical constituents subjected to the same analysis conditions. Thus, when there was a coincidence between retention times, the UV absorption spectrum was compared between the sample and standards, seeking to establish similarity [29].

A previously studied (in vitro and in vivo) concentration of 1% was used for the BRP dentifrice showing great antimicrobial and antibiofilm properties [13].

### 2.2. Analysis of Fluoride Stability in BRP Dentifrice

A total of three tubes of five different lots were selected among several produced batches by randomization and analyzed at different times (fresh, aged 12 months, aged 24 months). The tubes were coded with letters to enable a blind analysis. All the sample analyses were conducted in duplicate from each dentifrice tube. The nominal fluoride content of dentifrices was 1500 ppm F.

The initial analyses were carried out with fresh samples. Next, the dentifrice tubes were packed into a box and stored on a shelf for 24 months at room temperature (29 ± 2°C). The second analysis was performed after 12 months and the third after 24 months of storage (aged samples).

Fluoride was assayed according to the method of Cury et al. [30]. Dentifrices from each tube were analyzed using an ion-specific electrode (ISE) (Orion 96-09, Orion Research Inc., Beverly, EUA) previously calibrated with standard solutions of 100.00 μg/mL of fluoride (Analyze, Paraná, Brazil) ranging from 0.25 to 32.0 ppm F. The TF, TSF, and ionizable fluoride (IF) concentrations were determined, and the results were expressed in ppm F (μg F/g).

**Figure 1:**
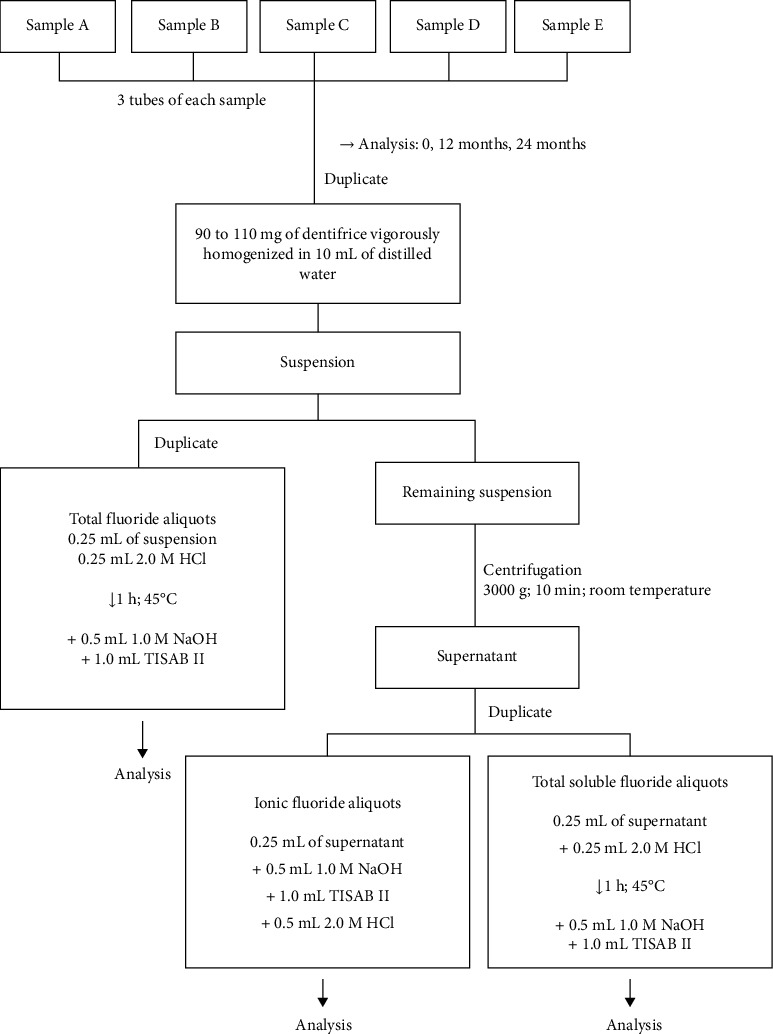
Analysis of fluoride stability in samples.

Blanks were used to desensitize the electrode between one reading and to act as an indicator of fluoride ion contamination. The curve pattern reading was performed in triplicate, and thus, the mean was obtained. An amount between 90 and 110 mg (± 0.01 mg) of each dentifrice was weighed and homogenized in 10 mL of distilled water. This procedure was repeated twice for each tube. Duplicates of 0.25 mL of the suspension were pipetted and transferred to test tubes to determine TF. The rest of the suspension was centrifuged (3000 g for 10 min at room temperature) to precipitate the insoluble fluoride that was attached to the abrasive. Duplicates of 0.25 mL of the supernatant were transferred to test tubes to determine the concentration of TSF and IF. Next, 0.25 mL of 2M HCl was added to the tubes for analysis of TF and TSF, and they were incubated at 45°C for 1 h. Then, all samples were neutralized with a volume of 0.5 mL of 1M NaOH and buffered with 1 mL of TISAB II. Lastly, 0.5 mL of 1 M NaOH, 1 mL of TISAB II, and 0.25 mL of 2 M HCl were added to the IF tubes for immediate reading (Figure 1).

### 2.3. Data Analysis

Millivolt potentials were converted to µg of fluoride using a standard curve with a correlation coefficient of *r* ≥ 0.99 and used to determine the F concentration in each dentifrice, expressed as ppm (µg F/g).

The results were initially analyzed by the Kolmogorov–Smirnov test to verify the distribution normality. Descriptive statistics were performed. Parametric analysis of variance (ANOVA) and paired *t*-tests were applied in the intragroup comparison to compare the means. The probability *α* of the type I error (significance level) was set at 0.05 (5%) in all cases, and a two-tailed *p*-value less than 0.05 was considered statistically significant. GraphPad Prism version 5.00 software for Windows (GraphPad Software, San Diego, California, USA, 2007) was used for both statistical procedures and graphing.

## 3. Results

Fluoride values in ppm can be seen in Tables 1, 2, and 3. The TF concentration of fresh and aged samples after 12 and 24 months of aging is shown in Table 1, the TSF concentration in Table 2, and the IF concentration in Table 3.

The TF after 12 months of storage was statistically significantly reduced in 60% of the samples (*p*  < 0.05). A reduction in TF in three (A, D, and E) of the five samples was found after 12 months of storage compared to the TF fresh (Table 1). After 24 months, in 60% of the samples, a significant reduction can be observed (*p*  < 0.05) compared to the previous 12 months (A, B, and E).

On Table 2, only sample B showed a significant reduction (*p*  < 0.05) regarding the TSF after 12 months of storage. All samples showed a significant reduction in TSF after 24 months of storage (Table 2).

A significant reduction in IF was found in 80% of the samples after 12 months of storage. All samples showed reduced IF after 24 months of storage (Table 3).

## 4. Discussion

The results of the present study show that the fluoride content of dentifrices was influenced by the storage time, being more critical with the storage period of 24 months. The fluoride content was within the expected standards in the fresh samples. After 12 months of storage, a significant reduction was observed in TF (3 samples) and IF (4 samples). Four samples (A, C, D, and E) showed stable values regarding TSF after 12 months of storage. Furthermore, most samples showed a significant reduction in TF, TSF, and IF after 24 months of storage (*p*  < 0.05).

The present study evaluated the concentration of different types of fluoride through a specific ion electrode, as well as in several studies reported in the literature [18, 19, 21–24, 26, 28–31] being the first one that analyzed the fluoride concentration in an experimental herbal toothpaste. Fresh BRP dentifrice samples showed TF values close to 1500 ppm F and TSF above 1000 ppm F. Even commercial dentifrices produced by large industries can present TF values below the declared, as found in the study by Ricomini Filho et al. [19] with the top 5 selling Brazilian dentifrices with different abrasives, which all dentifrices showed TF concentrations below 1500 ppm F and TSF concentrations above 1000 ppm F.

Storage conditions, temperature, and the natural aging process itself influence the soluble fluoride concentration in the dentifrice. The increase in temperature decreases the fluoride stability in dentifrice [25]. Therefore, it is important to evaluate aged samples in order to check if fluoride levels are maintained. In the present study, in which the samples were stored at room temperature (29 ± 2°C), most samples showed TF values close to 1500 ppmF and TSF close to 1000 ppm F after 12 months of storage. In other studies, the influence of these factors on fluoride concentrations is also perceived. In a study in Brazil, seven dentifrices were analyzed at the time of acquisition and after 12 months of storage at room temperature (28.9 ± 1.16°C) and under refrigerated air 26.3 ± 0.88°C. It was observed that the TSF in most dentifrices was not stable when stored, with the greatest loss occurring at room temperature and reaching values of 40%. In the dentifrices formulated with MFP/calcium carbonate (CaCO_3_), there was already 17% insoluble fluoride at the time of purchase. This increased to 40% after a year at room temperature, and it increased by 35% under refrigerated air. However, the TSF concentrations necessary to present anticaries potential were reached [31]; similar was observed in the study by Hashizume et al. [24] with the best selling dentifrices in Japan, analyzed at the time of purchase and after 1 year of storage at room temperature (21.8 ± 3.6°C). All dentifrices showed similar TF concentrations in fresh and aged samples according to Japanese legislation (content of less than 1000 ppm F). Dentifrices with silica as an abrasive showed stable fluoride concentrations. Some dentifrices with abrasive dicalcium phosphate showed a reduction in soluble fluoride concentration over time, forming insoluble fluoride [18–31]. However, all the analyzed dentifrices (fresh or aged) presented sufficient soluble fluoride concentrations to show anticaries activity. In the present study, due to the observed reduction in TF, it is assumed that part of the MFP may have reacted with propolis.

In a study by Fernandez et al. [23] a total of 30 dentifrices were analyzed in Chile after 1 year of storage at room temperature. The results showed that five presented between 30% and 50% of insoluble fluoride, four of which were formulated with MFP and calcium-based abrasive. Furthermore, a reduction in TSF below 1000 ppm F was found in two products [23]. This result is interesting, as it demonstrates the maintenance of appropriate amounts of TSF when stored for up to 12 months, especially those formulated with MFP and calcium-based abrasive.

The propolis dentifrice, despite the reduction in TSF, maintained values close to 1000 ppm F after 12 months of storage.

The results indicate that 24 months of aging led to a decrease in TF, TSF, and IF in the samples. The TSF, which has the potential to interfere in the caries process, was close to 1000 ppm F at 12 months of storage and below 1000 ppm F after 24 months of storage. A similar result was found in the study by Cury et al. [26] with the four best selling MFP/CaCO_3_ dentifrices in Brazil. These dentifrices maintain 1000 ppm of FST for a period of 1 year after manufacture; however, the TSF concentration close to the maturity date (36 months) was 44% lower, demonstrating that these dentifrices do not maintain a maximum desirable TSF concentration in this time.

Only one study that evaluated fluoride concentrations in commercial dentifrices incorporated with antimicrobial molecules was found. In this study, carried out in Brazil, it was observed that the dentifrices generally showed TF concentrations lower than those declared on the packaging; however, the TSF content remains close to 1000 ppm F [28].

Regarding the IF, the storage period led to a significant reduction. A few studies on the literature have evaluated the IF concentration, just in Chile and Brazil [27–31]. A similar result was observed in the study by Conde et al. [30], where the four dentifrices also based on MFP/CaCO_3_ showed a reduction in IF after 1 year of storage. This reduction occurs because the MFP undergoes hydrolysis over time and reacts with the calcium in the abrasive. It is noteworthy that only few studies in the literature have evaluated the IF present in dentifrices.

Brazilian regulation only requires that the TF content be declared on the dentifrice packaging (which must not exceed 1500 ppm F) [26]. Thus, the dentifrice manufacturers themselves do not assess or declare the fluoride content that can interfere with the caries process, the soluble fluoride.

The dentifrice evaluated in this study, which has already shown clinical and microbiological efficacy [13], demonstrates to maintain appropriate amounts of TSF when stored for up to 12 months. It is not a big problem since dentifrices have a high turnover in the establishments and it would hardly take more than a year to be sold. According to Conde et al. [31], the estimated time that dentifrices stay in a supermarket is ~3 months; thus, this time would be sufficient to prevent a loss of soluble fluoride. As it is an experimental dentifrice, few batches were analyzed in this study. Thus, more samples must be analyzed and in different storage conditions for greater quality control.

## 5. Conclusion

In conclusion, the fluoride values in fresh samples were as expected; however, the fluoride content was influenced by the storage time. The TSF values remained relatively stable after 1 year of aging; however, 2 years of aging of the dentifrice led to a reduction in TF, TSF, and IF.

## Figures and Tables

**Table 1:** Average and standard deviation of the samples in relation to the total fluoride of the fresh and aged samples.

Sample	TF fresh sample Mean ± SD (ppm F)	TF 12 months Mean ± SD (ppm F)	TF 24 months (ppm F)
A	1410.6 ± 26.9	1278.6 ± 88.3*⁣*^*∗*^	1083.1 ± 32.4^十^
B	1441.7 ± 151.1	1241.8 ± 21.9	820.4 ± 192.8^十^
C	1473.5 ± 108.5	1443.6 ± 33.4	1037.4 ± 283.3
D	1408.1 ± 29.3	1198.9 ± 163.3*⁣*^*∗*^	1000.9 ± 254.1
E	1409.9 ± 68.9	1320.9 ± 30.2*⁣*^*∗*^	763.5 ± 73.2^十^

Abbreviation: TF, total fluoride

*⁣* ^*∗*^ *p* < 0.05 denotes statistical difference in the TF fresh samples in relation to TF 12 months.

^十^ *p*  < 0.05 denotes statistical difference in the TF 12 months in relation to TF 24 months.

**Table 2:** Average and standard deviation of the samples in relation to the total soluble fluoride of the fresh and aged samples.

Sample	TSF fresh sample Mean ± SD (ppm F)	TSF 12 months Mean ± SD (ppm F)	TSF 24 months Mean ± SD (ppm F)
A	1031.6 ± 59.1	934.2 ± 77.5	758.4 ± 141.1^十^
B	1000.3 ± 33.4	912.0 ± 53.9*⁣*^*∗*^	562.1 ± 72.3^十^
C	1059.4 ± 50.1	869.6 ± 105.5	556.9 ± 38.3^十^
D	1065.0 ± 11.0	965.8 ± 149.8	552.3 ± 39.9^十^
E	1039.3 ± 77.4	925.1 ± 81.7	588.1 ± 53.0^十^

Abbreviation: TSF, total fluoride

*⁣* ^*∗*^ *p*  < 0.05 denotes statistical difference in the TSF fresh samples in relation to TSF 12 months.

^十^ *p*  < 0.05 denotes statistical difference in the TSF 12 months in relation to TF 24 months.

**Table 3:** Average and standard deviation of the samples in relation to the ionizable fluoride of fresh and aged samples.

Sample	IF fresh sample Mean ± SD (ppm F)	IF 12 months Mean ± SD (ppm F)	IF 24 months Mean ± SD (ppm F)
A	387.9 ± 55.1	269.7 ± 10.1*⁣*^*∗*^	150.2 ± 8.4^十^
B	359.9 ± 10.1	223.5 ± 14.8*⁣*^*∗*^	174.7 ± 4.9^十^
C	352.7 ± 7.6	246.8 ± 33.1*⁣*^*∗*^	102.2 ± 4.0^十^
D	319.3 ± 56.5	242.0 ± 26.8*⁣*^*∗*^	130.2 ± 7.6^十^
E	335.5 ± 63.8	253.3 ± 35.1	142.1 ± 8.1^十^

Abbreviation: IF, total fluoride.

*⁣* ^*∗*^ *p*  < 0.05 denotes statistical difference in the IF fresh samples in relation to IF 12 months.

^十^ *p*  < 0.05 denotes statistical difference in the IF 12 months in relation to IF 24 months.

## Data Availability

The authors confirm that the data supporting the findings of this study are available within the article, and upon reasonable request, raw data will be made available by the corresponding author.
